# Development and validation of a specific scale on exercise compliance of lumbar disc herniation patients with conservative treatment

**DOI:** 10.3389/fpubh.2025.1671694

**Published:** 2025-10-08

**Authors:** Caixiang Gao, Xueyan Huang, Lu Han, Jingying Zhang, Rui Wang, Guijuan He

**Affiliations:** ^1^Massage Department, Hangzhou Hospital of Traditional Chinese Medicine, Affiliated to Zhejiang University of Traditional Chinese Medicine, Hangzhou, China; ^2^Zhejiang University of Traditional Chinese Medicine, Hangzhou, China

**Keywords:** lumbar disc herniation, conservative treatment, exercise compliance, scale development, reliability, validity

## Abstract

**Background:**

We aim to develop a specific scale on exercise compliance of patients with lumbar disc herniation undergoing conservative treatment and to evaluate its reliability and validity.

**Methods:**

The scale was developed in two stages. In the first stage, a preliminary version of the scale was developed through literature review, research group discussions, Delphi expert consultations, and pilot testing, based on the Health Belief Model and the concept framework of rehabilitation training compliance. In the second stage, the reliability and validity of the scale were tested among 430 patients with lumbar disc herniation undergoing conservative treatment who were discharged from the Massage Department of a tertiary hospital in Zhejiang Province within 2 months from May to August 2023. They were selected with a convenience sampling method.

**Results:**

The developed scale on exercise compliance included four dimensions: preparation compliance, exercise compliance, supervision compliance, and recommendation compliance, with a total of 20 items. The content validity index for the scale and each item was 0.914 and 0.813 to 1.000, respectively. An exploratory factor analysis extracted four common factors with a cumulative variance contribution rate of 73.578%. The results of confirmatory factor analysis showed a good model fit, with a chi-square/degree of freedom ratio of 2.642, incremental fit index of 0.929, comparative fit index of 0.928, and root mean square error of approximation of 0.069. The scale’s Cronbach’s *α* coefficient was 0.942, split-half reliability was 0.866, and test–retest reliability was 0.901.

**Conclusion:**

The development process of this exercise compliance scale was scientifically rigorous. The scale exhibits excellent reliability and validity, making it an effective tool for assessing exercise compliance in LDH patients receiving conservative treatment.

## 1 Introduction

Lumbar disc herniation (LDH) is a common condition characterized by the degeneration of the lumbar intervertebral disc, rupture of the annulus fibrosus, and protrusion of the nucleus pulposus tissue, which stimulates or compresses the lumbar and sacral nerve roots and the cauda equina nerve (1, 2). It is one of the common causes of low back pain. According to the Global Burden of Disease Study 2021, low back pain affects approximately 619 million people worldwide and is a major contributor to disability and rehabilitation needs related to musculoskeletal disorders (3, 4).

Treatment methods for LDH currently mainly include surgical treatment and conservative treatment (2). Conservative treatment is the preferred treatment for LDH patients without significant nerve damage (1). Exercise therapy, including core muscle strength training, lumbar stability exercises, back functional muscle training, Pilates, Tai Chi, and other mind–body exercises, has shown good therapeutic effects (5). It is recommended as an effective intervention measure in European and American clinical practice guidelines (6). A key principle emphasized in The Lancet Low Back Pain series is the use of a biopsychosocial framework to guide management, which includes functional exercises, health education, self-management, and resumption of daily activities (7–9). Therefore, adherence to a supervised therapeutic exercise plan is crucial for effective conservative management of LDH (10), and accurately measuring patient compliance is essential for evaluating the effectiveness of functional exercise interventions (11).

The functional exercise compliance scales, including generic scales and specific scales, have been developed. Generic scales, such as the sports injury rehabilitation adherence scale (12), the exercise adherence rating scale (13), and the exercise adherence scale (14), lack specificity in evaluating functional exercise adherence in conservative treatment of LDH patients. Specific scales for LDH patients include the functional exercise adherence scale for orthopedic patients (15), the orthopedic functional exercise adherence scale (16), and the postoperative functional exercise adherence scale for LDH patients (17), mainly targeting surgical patients. However, these scales are not applicable for evaluating patients with conservative treatment. Currently, there is still a lack of evaluation tools with good reliability and validity for assessing functional exercise adherence in LDH patients with conservative treatment.

The Health Belief Model (HBM) (18) was initially proposed by Hochbaum in 1958 and has been widely used in health behavior research related to compliance. The model emphasizes cognitive factors, including perception of susceptibility to disease, perception of severity of disease, perception of benefits of adopting healthy behaviors, perception of barriers to adopting healthy behaviors, perception of factors promoting healthy behaviors, and self-efficacy, which are essential drivers of individual behavior change. The concept framework of rehabilitation training compliance (19) suggests that patient participation in prescribed exercise plans should be measured in terms of frequency, duration, intensity, correctness, and disease specificity. These two compliance models are widely used in guiding the application of the scale of exercise compliance.

Herein, based on the HBM and the concept framework of rehabilitation training compliance, this study developed a specific compliance measurement scale for functional exercises in LDH patients with conservative treatment. Its reliability and validity were evaluated. This scale may provide an effective measurement tool for assessing functional exercise compliance and a theoretical basis for the development of compliance strategies in LDH patients with conservative treatment. It may also serve as a reference and guide for healthcare professionals in providing extended care at different stages.

## 2 Methods

### 2.1 A preliminary version of the specific scale on functional exercise compliance of patients with LDH undergoing conservative treatment

#### 2.1.1 Research team

The research team consisted of 7 members, including 1 graduate supervisor, 1 doctor of acupuncture and massage, 1 chief physician, 2 senior nursing experts in massage therapy, and 2 nursing graduate students. The graduate supervisor was responsible for the overall project management, while medical and nursing experts conducted literature research, analyzed expert inquiries and statistical results, and suggested modifications to scale items. Graduate students were tasked with managing literature research, expert inquiries, scale, and data collection.

#### 2.1.2 Establishment of the initial item pool

The item pool was established through a dual foundation of theoretical modeling and literature synthesis.

Theoretical basis: after discussions within the research group, based on the HBM, and referencing the Exercise Adherence Rating Scale (13), the Compliance Scale for Functional Exercises in Orthopedic Patients (15), the Compliance Scale for Rehabilitation Training Following Total Knee Arthroplasty (20), and the established compliance measurement scales related to stroke, we tentatively identified four dimensions for constructing the compliance scale for functional exercises: physical participation-related compliance for exercises, supervision-related compliance for exercise effects, active advice-seeking during exercise, and preliminary preparation-related compliance for functional exercises. Furthermore, the Concept Framework of Rehabilitation Training Compliance informed the wording of the items to include descriptions of “consistency in doing,” “daily degree of adherence,” and “strict adherence to requirements.”

Literature review: A systematic search was conducted in Chinese databases (CNKI, Wanfang, Chinese Biomedical Literature Database) and English databases (PubMed, Embase) to collect relevant literature on LDH rehabilitation exercises and existing compliance scales. This study generated an initial pool of 21 potential items that aligned with the theoretical dimensions.

#### 2.1.3 Delphi expert consultation process

A formal two-round Delphi process was employed to refine the scale items and assess content validity.

Expert Panel: From November 2022 to February 2023, we invited 18 experts from Zhejiang Province, China. Experts were selected based on the following criteria: (1) a minimum of 10 years of work experience in rehabilitation, orthopedics, acupuncture, massage, or nursing; (2) a Bachelor’s degree or higher; (3) an intermediate professional title or higher; and (4) willingness to participate actively in both rounds of consultation.

Procedure: The expert consultation package consisted of: (1) a cover letter explaining the study’s purpose and Delphi requirements; (2) a form to collect demographic information (such as gender, age, research field, years of work, professional title, highest education level, and other basic details); (3) a form to assess the expert’s familiarity with the topic and their judgment basis; and (4) the preliminary scale for evaluation.

Evaluation and Item Screening: Experts rated each item on a 5-point Likert scale (ranging from “very unimportant” to “very important” with scores of 1–5, and from “very unfeasible” to “very feasible” with scores of 1–5) for its importance and feasibility. The predetermined criteria for item retention were an average score > 3.50 for both importance and feasibility, and a coefficient of variation <0.25 (21). Based on qualitative feedback and research team discussions, modifications were made to dimensions and items between rounds. The consultation concluded after two rounds when expert opinions reached consensus. The final version after Delphi consisted of 4 dimensions and 21 items.

#### 2.1.4 Pilot testing

A pilot test was conducted to assess the clarity, acceptability, and initial performance of the scale items.

Sample: A convenience sample of 90 LDH patients who underwent conservative treatment and were discharged from the massage department of a tertiary hospital in Zhejiang Province during March–April 2023 was selected (using the same inclusion and exclusion criteria as the formal survey).

Procedure: Patients were briefed on the study’s purpose and provided informed consent. Then, researchers administered the preliminary 21-item scale. Participants were asked to complete the scale based on their functional exercise practices over the preceding 2 weeks. The primary objectives of this pilot phase were to: (1) identify any items that were ambiguous, difficult to understand, or potentially misleading to the target population; (2) evaluate the average time required for completion; and (3) gather initial feedback on the overall acceptability and face validity of the scale.

Analysis and Refinement: Data collected from the pilot test were subjected to preliminary analysis using the same statistical methods planned for the formal study (i.e., discrete trend analysis, critical ratio (CR) analysis, and correlation coefficient analysis) to identify any items with potential performance issues. More importantly, qualitative feedback on item comprehension and relevance was collected informally from participants upon completion of the scale. This feedback was reviewed and discussed extensively by the research team. Based on this combined quantitative and qualitative assessment, minor refinements were made to the wording and phrasing of several items to enhance clarity and ensure all instructions and items were universally intelligible. The pilot testing confirmed the feasibility of the scale and resulted in a preliminary version with 4 dimensions and 21 items ready for large-scale validation.

### 2.2 Reliability and validity testing of the scale

#### 2.2.1 Study participants

LDH patients, who had received conservative treatment and were discharged from the massage department of a tertiary hospital in Zhejiang Province between May and August 2023, were selected using a convenience sampling method. Patients were included in the study if they met all of the following criteria: (1) Patients aged 18 years or older; (2) Patients diagnosed with LDH according to relevant diagnostic criteria (22) and confirmed by imaging examinations such as X-ray or magnetic resonance imaging; (3) Patients underwent conservative treatment (e.g., manual therapy, acupuncture, physical therapy, medication) as their primary management strategy; (4) Patients were within 2 months of being discharged from the hospital department; (5) Patients were conscious, informed about the study, and voluntarily provided written informed consent to participate. Patients were excluded from the study if they met any of the following criteria: (1) Patients with concomitant tumors or spinal metastases; (2) Patients with concomitant severe neurological (e.g., stroke, Parkinson’s disease), muscular, or metabolic disorders (e.g., uncontrolled diabetes) that could significantly impair their ability to perform functional exercises; (3) Patients had a history of spinal surgery; (4) Patients presented with clinical signs of cauda equina syndrome (e.g., bowel or bladder dysfunction, significant saddle anesthesia) or had significant muscle atrophy or paralysis; (5) Pregnant or lactating women; (6) Patients with lumbar spondylolisthesis or lumbar spine tuberculosis; (7) Patients who had cognitive impairments or communication barriers that prevented them from understanding or completing the questionnaire. This study has been approved by the Ethics Committee of Zhejiang University of Traditional Chinese Medicine (Approval No: 2020KY080). All methods were performed in accordance with the relevant guidelines and regulations. All participants signed the written informed consent.

#### 2.2.2 Sample size justification

The sample size for this study was determined based on the requirements for conducting factor analysis, which is the primary statistical method for validating scale structure. A widely accepted rule of thumb in scale development is that the sample size should be 5–10 times the number of items on the scale (23). Our preliminary scale consisted of 21 items after the Delphi process. Thus, a minimum sample size of 210 (10 × 21) was required. Furthermore, for confirmatory factor analysis, a sample size of at least 200 is generally considered the bare minimum to obtain stable parameter estimates (23). To ensure the robustness of our analysis, account for potential invalid or incomplete responses, and enhance the generalizability of our findings, we increased the target sample size. Considering an estimated invalid response rate of 10–20%, a final sample size of 430 was determined to be adequate to meet the statistical requirements and ensure the validity of the study results.

#### 2.2.3 Survey tools

The general information survey scale included 11 items, i.e., gender, age, height, marital status, living arrangements, education level, medical payment methods, family income, duration of illness, main symptoms, and whether they have other chronic diseases. The preliminary version of a functional exercise compliance scale for patients with LDH with conservative treatment consisted of 4 dimensions and 21 items. It used a Likert 5-point scoring method with response options: “completely unable to do,” “basically unable to do,” “sometimes able to do,” “basically able to do,” and “completely able to do,” scored as 1, 2, 3, 4, and 5, respectively. The score for each dimension was the total score of items in that dimension, with higher scores indicating greater compliance demonstrated by patients in that dimension.

#### 2.2.4 Data collection methods

In strict accordance with inclusion and exclusion criteria, participants were selected, and data were collected through a combined approach of on-site and online surveys. For on-site surveys, two research graduates who received unified training explained the reasons and purposes of the survey to the patients using standardized instructions and questioning methods, ensuring the confidentiality of survey information. Online surveys were conducted by the researcher (YYY), who developed an electronic version of the questionnaire, which was reviewed by the research team before being self-administered by the survey participants, with an IP recognition function enabled. Following the completion of questionnaire collection, two research team members promptly screened the data and removed any invalid questionnaires. The exclusion criteria included answers exhibiting obvious patterns or logical inconsistencies, and completion time for the electronic questionnaire of less than 5 min.

#### 2.2.5 Selection of scale items

Item selection was performed using the discrete trend analysis, CR analysis, and correlation coefficient analysis to improve the sensitivity and stability of the scale items. For the discrete trend analysis, items with a standard deviation of less than 0.75 were excluded. In the CR analysis, the total scale scores were divided into the top 27% (high-score group) and the bottom 27% (low-score group). Independent sample t-tests were performed to compare the differences in item scores between these two groups, with items showing CR values less than 3 or no statistically significant differences (*p* > 0.05) being deleted. In the correlation coefficient analysis, the relevance of each questionnaire item to the total questionnaire was evaluated, and items with correlation coefficients that were not statistically significant (*p* > 0.05) or less than 0.4 were removed (24).

#### 2.2.6 Validity test

Content validity: the item-level content validity index (I-CVI) and the scale-level overall content validity index (S-CVI) were calculated based on ratings from the expert panel. Experts rated the relevance of each item on a 5-point scale: 1 = very irrelevant, 2 = irrelevant, 3 = somewhat relevant, 4 = quite relevant, 5 = highly relevant. I-CVI was calculated as the number of experts giving a rating of 4 or 5, divided by the total number of experts. The S-CVI was calculated as the average of all I-CVIs. An I-CVI > 0.70 and an S-CVI > 0.80 indicate good content validity of the scale (25).

Construct validity: Both Exploratory Factor Analysis (EFA) and Confirmatory Factor Analysis (CFA) were conducted to evaluate the construct validity of the scale. EFA was performed on a randomly selected half of the sample (*n* = 206) using SPSS 27.0. The suitability of data for EFA was assessed using the Kaiser-Meyer-Olkin measure and Bartlett’s test of sphericity. Principal component analysis and maximum likelihood extraction were used for factor analysis, with factors extracted based on eigenvalues ≥1, cumulative variance contribution rate >50%, and at least 3 items per factor according to the scree plot. Items with factor loadings <0.45, multiple high loadings with values close to each other (difference <0.2), or improperly categorized and difficult to explain were deleted. CFA was performed on the second half of the sample (*n* = 206) using AMOS 28.0. CFA involved model analysis using maximum likelihood estimation to confirm the construct appropriateness and stability of the theoretical structure of the scale. A chi-square to degrees of freedom ratio (χ^2^/df) of 1.0–3.0 indicates a good fit between the hypothesized model and the sample data. Additionally, a Root Mean Square Error of Approximation (RMSEA) < 0.08, Comparative Fit Index (CFI) > 0.90, Incremental Fit Index (IFI) > 0.90, and Tucker-Lewis Index (TLI) > 0.90 suggest a good model fit. The scale was considered to have good discriminant validity if the square root of the average variance extracted (AVE) of the dimension was greater than the correlation coefficient between this dimension and other dimensions (26). The internal correlation test involved calculating Pearson correlation coefficients to assess the relationship between items and their respective dimensions, among dimensions, and between dimensions and the total scale.

#### 2.2.7 Reliability test

Internal consistency was evaluated using Cronbach’s alpha coefficient and the split-half reliability coefficient. A Cronbach’s *α* coefficient >0.80 for the overall scale and >0.70 for each dimension, as well as a split-half reliability >0.80, indicate good internal consistency of the scale (27). Test–retest reliability was assessed to evaluate the stability of the scale over time. A subset of participants (*n* = 30) was asked to complete the scale again after a three-week interval. A test–retest reliability >0.70 indicates good stability of the scale (28).

### 2.3 Statistical methods

Statistical analysis was conducted using SPSS 27.0 (SPSS Inc., Chicago, IL, USA) and AMOS 28.0 (IBM Corp., Armonk, NY, USA). Normally distributed measurement data are presented as mean ± standard deviation, while non-normally distributed data are presented as median (P25, P75). Categorical data are described using frequency and composition ratios. The enthusiasm of experts was evaluated using the expert enthusiasm coefficient, the authority of experts was assessed using the expert authority coefficient, and the consensus among expert opinions was measured using Kendall’s coefficient of concordance. A significance level of *p* < 0.05 was used to indicate the statistical significance of differences.

## 3 Results

### 3.1 Expert consultation results

In this study, a total of 18 experts from Zhejiang were invited to participate in the consultation. Sixteen experts completed two rounds of consultations, with an average age of (46.81 ± 8.74) years old. Among them, 4 experts held intermediate titles, 5 held associate senior titles, and 7 held full senior titles. The average years of work experience were (23.50 ± 11.02) years, including 10 acupuncture and massage doctors and 6 nurses with orthopedic rehabilitation nursing experience. The experts’ participation rates in the two rounds of consultations were 88.89 and 100%, respectively. The coefficient of evaluation basis for the experts was 0.994, the familiarity coefficient was 0.975, and the authority coefficient was 0.985, indicating high enthusiasm and authority among the experts, making the consultation results reliable. The Kendall’s concordance coefficient for importance was 0.196 (χ^2^ = 75.152) and 0.219 (χ^2^ = 84.117) respectively, while for feasibility they were 0.143 (χ^2^ = 54.955) and 0.218 (χ^2^ = 83.599) respectively. All coefficients were statistically significant at *p* < 0.001, indicating good concordance of expert opinions and the reliability of the results.

In the first round of expert consultations, based on their opinions, the research team deleted one secondary item, merged one secondary item, and added two new secondary items after discussion. In the second round of expert consultations, the importance ratings of the scale items ranged from 3.75 to 4.88 with a coefficient of variation of 0.07–0.23, and the feasibility ratings ranged from 3.75 to 4.81 with a coefficient of variation of 0.08–0.22. No new objections were raised by the experts regarding the dimensions and item content, and a suggestion from one expert to change the order of dimensions in the scale was accepted.

### 3.2 Results of item selection

The analysis using the discrete trend method showed that the standard deviations of all items were greater than 0.75, indicating good dispersion trends of the scale. The CR method reveals CR values ranging from 7.95 to 27.50 for each item, with statistically significant differences between items (P all < 0.001), suggesting good discriminatory power of the scale. The analysis using the correlation coefficient method revealed that item Q3 “Avoid dangerous movements such as bending over, lifting heavy objects excessively, getting chilled, wearing tight pants, and wearing high heels as advised by healthcare providers” had a correlation coefficient of less than 0.4 with the total score, suggesting deletion. However, after discussion by the research team, it was considered important for the compliance dimension of pre-preparation, which can alert patients to disease precautions. Therefore, this item was retained for further analysis. The correlation coefficients between other items and the total score range from 0.468 to 0.876, indicating that the correlation between items and the total score of the scale is within an acceptable range.

### 3.3 Results of the reliability and validity testing of the scale

#### 3.3.1 General information about the survey and the study participants

A total of 430 questionnaires were distributed in this survey, all of which were returned. After excluding 18 invalid questionnaires, 412 valid questionnaires were collected, resulting in a response rate of 100% and an effective rate of 95.81%. The general information of the study participants is presented in Table 1.

**Table 1:** The general information of the study participants (*n* = 412).

Variables	Number of cases	Percentage (%)	Variables	Number of cases	Percentage (%)
Gender			Per capita monthly household income		
Male	186	45.1	≤3,000	39	9.5
Female	226	54.9	3,001–4,000	82	19.9
Age (year)			4,001–5,000	97	23.5
≤30	42	10.2	5,001–6,000	116	28.2
31–40	91	22.1	≥6,001	78	18.9
41–50	91	22.1	Body mass index (kg/m^2^)		
51–60	89	21.6	<18.5 (thin)	66	16.0
61–70	71	17.2	18.5 ~ (Normal)	212	51.5
≥71	28	6.8	24.0 ~ (overweight)	97	23.5
Marriage status			28.0 ~ (obese)	37	9.0
Single	52	12.6	Duration of disease (years)		
Married	307	74.5	<5	200	48.5
Divorced	40	9.7	5~	106	25.7
Widowed	13	3.2	10~	78	18.9
Highest Education Level			20~	28	6.8
Primary school or below	34	8.3	Clinical symptoms		
Junior high school	78	18.9	Only pain symptoms	162	39.3
High school (including vocational school)	97	23.5	Only numbness and weakness symptoms	109	26.5
College degree or above	203	49.3	Two or more symptoms	134	32.5
Healthcare Payment Method			Other	6	1.5
Medical insurance (urban, new rural cooperative medical care, commercial insurance, etc.)	345	83.7	Concomitant disease		
Free medical service	39	9.5	None	293	71.1
Fully self-funded	28	6.8	One Concomitant disease	88	21.4
Living arrangement			Two or more concomitant diseases	31	7.5
Living alone	84	20.4			
Living only with a spouse	90	21.8			
Living with spouse and children or parents	238	57.8			

#### 3.3.2 Content validity

The content validity of the scale was excellent. The I-CVI for the items ranged from 0.813 to 1.000 (all exceeding the 0.70 criterion), and the S-CVI was 0.914 (exceeding the 0.80 criterion).

#### 3.3.3 Construct validity

**Figure 1:**
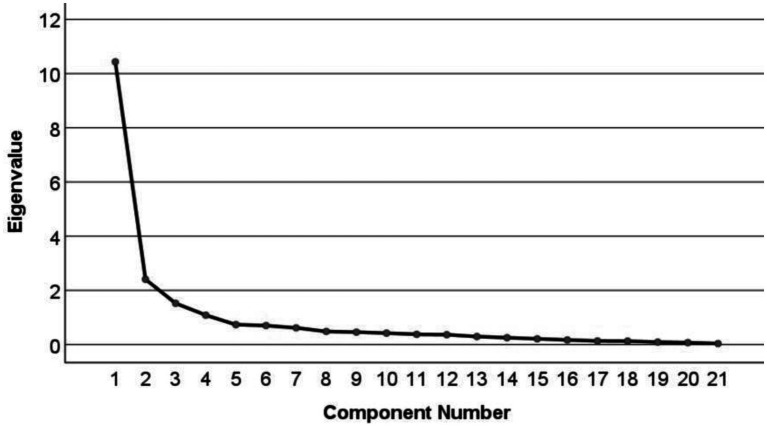
Scree plot of factor analysis.

EFA revealed that the Kaiser-Meyer-Olkin value was 0.862 and Bartlett’s sphericity test was significant (χ^2^ = 3967.718, *p* < 0.001), indicating that the data were suitable for factor analysis (29). Four factors with eigenvalues greater than 1 were extracted, accounting for a cumulative variance of 73.578%. The scree plot (Figure 1) showed a clear inflection point after the fourth common factor, indicating a basic agreement between the four common factors and the dimensionality hypothesis of the scale. The factor loading matrix for each item after rotation (Table 2) revealed that all item factor loadings were >0.55. Item Q2 “Start functional exercise as early as the condition permits” loaded 0.554 on Factor 3 and 0.558 on Factor 4, showing a situation of multiple loadings. However, upon discussion within the research team, it was decided to retain this item as it was considered crucial for functional exercise in LDH patients and indispensable for the construction of the scale. On the other hand, Item Q17 “Can undergo regular follow-up examinations, communicate with healthcare providers about the effectiveness of the functional exercise program, and make adjustments as needed” did not align with its originally assigned factor (23). After deliberation within the research team, Item Q17 was removed. Finally, a functional exercise compliance scale for LDH patients with conservative treatment, comprising four dimensions and 20 items, was developed.

**Table 2:** The factor loading matrix for each item after rotation (*n* = 206).

Items	Factor loading			
	Exercise compliance	Recommendation compliance	Supervision compliance	Preparation compliance
Q1: Receive a personalized functional exercise program designed by healthcare providers for you	0.215	0.254	0.143	0.710
Q2: Start the functional exercise as early as the condition permits	0.238	0.142	0.554	0.558
Q3: Avoid dangerous movements such as bending over, lifting heavy objects excessively, getting chilled, wearing tight pants, and wearing high heels, as advised by healthcare providers	0.191	0.049	−0.169	0.833
Q4: Can master the specific methods of LDH functional exercise	0.115	0.271	0.513	0.617
Q5: Can do supine leg raise exercises every day	0.590	0.147	0.190	0.241
Q6: Can persevere in doing bridge exercises every day	0.835	0.179	0.140	0.170
Q7: Can persist in doing push-ups exercise every day	0.869	0.033	0.038	0.151
Q8: Can adhere to doing the reverse fly exercise every day	0.823	0.146	0.191	0.217
Q9: Can persevere in doing supine knee flexion exercises every day	0.733	0.105	0.232	0.173
Q10: Can persevere in doing knee-hand balance exercises every day	0.828	0.124	0.207	0.111
Q11: Can strictly follow the exercise frequency requirements in the program for functional exercise every day	0.731	0.154	0.494	0.089
Q12: Can strictly adhere to the exercise duration requirements in the program for functional exercise every time	0.758	0.202	0.494	0.070
Q13: Can follow the requirements of medical staff without omitting any exercise in the functional exercise program	0.726	0.143	0.480	0.068
Q17: Can undergo regular follow-up examinations, communicate with healthcare providers about the effectiveness of the functional exercise program, and make adjustments as needed	0.673	0.446	0.144	0.059
Q14: Can independently schedule an appropriate time for functional exercise	0.433	0.182	0.638	0.047
Q15: Can actively observe and evaluate the effects of functional exercise	0.491	0.231	0.687	0.057
Q16: Under the encouragement and supervision of those around them (including family, friends, or fellow patients), can persist in following the requirements of functional exercise	0.335	0.386	0.700	0.014
Q18: Can seek timely consultation from healthcare providers when facing problems that cannot be resolved by themselves or their family	0.068	0.851	0.082	0.089
Q19: Can actively seek advice from healthcare providers when not achieving the expected exercise results	0.185	0.764	0.364	0.090
Q20: Can proactively seek help from healthcare providers when experiencing back or lower limb pain during exercise	0.185	0.833	0.172	0.206
Q21: Pathways for seeking help are unobstructed	0.196	0.826	0.108	0.162
Characteristics	6.689	3.467	3.097	2.199
Contribution rate (%)	31.852	16.509	14.747	10.471
Cumulative variance contribution rate (%)	31.852	48.36	63.107	73.578

**p < 0.001. LDH, lumbar disc herniation.

**Figure 2:**
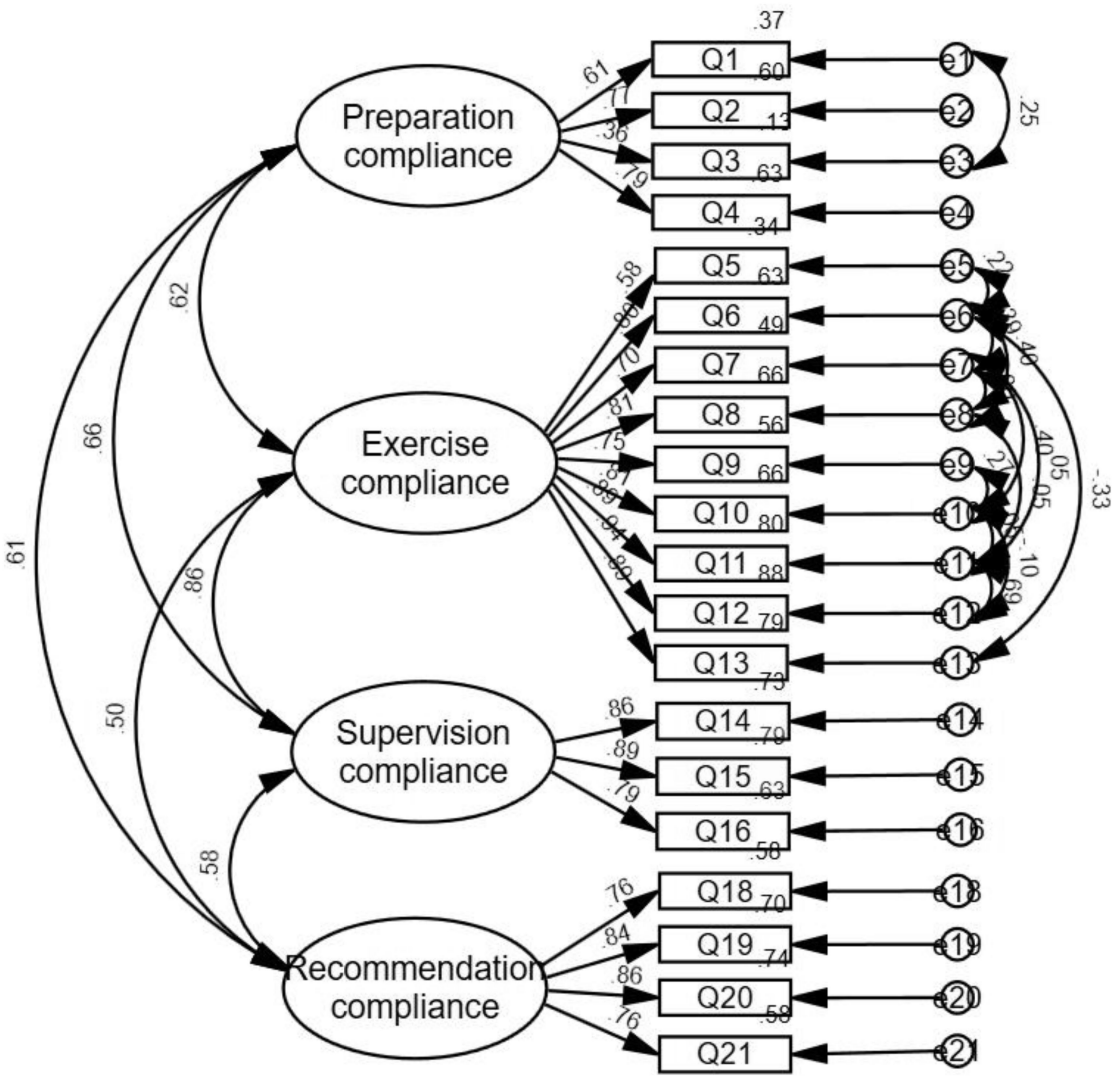
Model fit graph of the revised functional exercise compliance scale for LDH patients with conservative treatment.

In the CFA, the initial model fit was inadequate (χ^2^/df = 4.627, RMSEA = 0.133, IFI = 0.830, TLI = 0.801, CFI = 0.828, and RMR = 0.073), indicating that further modifications were needed. After adjusting by adding error terms e1 with e3, e5 with e6, e6 with e7, e8, and e13, e7 with e8, e10, and e11, e8 with e11, e9 with e10, e10 with e11 and e12, and, e11 with e12 covariances, the model fit improved significantly. The final model fit indices were: *χ*^2^/df = 2.642, RMSEA = 0.069, IFI = 0.929, TLI = 0.910, CFI = 0.928, and RMR = 0.061. All standardized factor loadings were statistically significant (*p* < 0.001) and exceeded 0.6, demonstrating strong relationships between the items and their respective latent constructs. Figure 2 illustrates the model fit after modification.

#### 3.3.4 Discriminant validity

The square roots of the AVE values for preparation compliance, exercise compliance, supervision compliance, and recommendation compliance were 0.805, 0.846, 0.804, and 0.657, respectively (Table 3). The square root of the AVE for the supervision compliance dimension (0.846) was slightly smaller than 0.861, while the square roots of the AVEs for the other dimensions were larger than the correlation coefficients between that dimension and the other dimensions, indicating that the scale has discriminant validity.

**Table 3:** The discriminant validity of the functional exercise compliance scale for LDH patients with conservative treatment.

Variables	Recommendation compliance	Supervision compliance	Exercise compliance	Preparation compliance
Recommendation compliance	0.649			
Supervision compliance	0.583	0.716		
Exercise compliance	0.497	0.861	0.647	
Preparation compliance	0.613	0.663	0.621	0.432
The square root of the AVE	0.805	0.846	0.804	0.657

LDH, lumbar disc herniation; AVE, average variance extracted.

#### 3.3.5 Internal correlation analysis

The correlation coefficients between the dimensions ranged from 0.424 to 0.743, indicating a moderate to low correlation (Table 4). The correlation coefficients between each dimension and the total scale ranged from 0.675 to 0.927, indicating a moderate to high correlation (all *p* < 0.001).

**Table 4:** The internal correlation of the functional exercise compliance scale for LDH patients with conservative treatment.

Variable	Preparation compliance	Exercise compliance	Supervision compliance	Recommendation compliance	Total score of the scale
Preparation compliance	1.000	—	—	—	—
Exercise compliance	0.516**	1.000	—	—	—
Supervision compliance	0.464**	0.743**	1.000	—	—
Recommendation compliance	0.461**	0.424**	0.534**	1.000	—
Total score of the scale	0.699**	0.927**	0.838**	0.675**	1.000

**p < 0.001. LDH, lumbar disc herniation.

#### 3.3.6 Reliability

The reliability coefficients for the overall scale and each dimension are summarized in Table 5. The overall scale exhibited excellent internal consistency, with a Cronbach’s *α* of 0.942. The Cronbach’s α values for the dimensions ranged from 0.772 to 0.947. The split-half reliability was 0.876 for the overall scale, while the dimensional split-half reliability ranged from 0.761 to 0.914. Test–retest reliability, assessed in 30 patients after a three-week interval, was 0.901 for the overall scale and ranged from 0.821 to 0.910 for the dimensions (all *p* < 0.001), indicating good stability of the scale over time and high test–retest reliability.

**Table 5:** The reliability of the functional exercise compliance scale in LDH patients with conservative treatment.

Reliability coefficients		Cronbach’s α coefficient	Split-half reliability	Test–retest reliability
Total scale		0.942	0.876	0.901
Dimensions	Preparation compliance	0.772	0.761	0.821**
	Exercise compliance	0.947	0.914	0.859**
	Supervision compliance	0.873	0.841	0.896**
	Recommendation compliance	0.888	0.862	0.910**

**p < 0.001. LDH, lumbar disc herniation.

## 4 Discussion

### 4.1 Key findings and comparative advantage

This study, supported by the HBM and the concept framework of rehabilitation training compliance, constructed a scale consisting of 4 dimensions (preparation compliance, exercise compliance, supervision compliance, and recommendation compliance) and 20 items. The development process was rigorous, and the final scale demonstrates excellent psychometric properties, with strong reliability (Cronbach’s *α* = 0.942, test–retest reliability = 0.901) and validity (S-CVI = 0.914, good model fit indices), indicating that it is a robust and scientifically sound tool for measuring exercise compliance in conservatively treated LDH patients.

Our scale addresses a significant gap in the existing measurement landscape. While generic adherence scales exist (12–14), they lack specificity for LDH and fail to capture unique aspects of conservative management. Similarly, existing LDH-specific scales (15–17) focus predominantly on post-surgical rehabilitation, including items about surgical wounds and inpatient therapy that render them inappropriate for non-operative patients. Our scale is the first specifically designed for the conservative treatment pathway, filling this critical measurement gap.

### 4.2 In-depth interpretation of the four-dimensional structure

The scale contains four interconnected domains. The ‘Preparation Compliance’ dimension assesses foundational knowledge and behaviors prerequisite to exercise (e.g., receiving a personalized plan, avoiding risky movements), aligning with the ‘perceived susceptibility’ and ‘severity’ constructs of the HBM. The ‘Exercise Compliance’ dimension measures the core performance of prescribed exercises. The ‘Supervision Compliance’ dimension evaluates the role of external support and self-monitoring, enhancing ‘self-efficacy’ (HBM). Finally, the ‘Recommendation Compliance’ dimension captures proactive help-seeking behavior, which facilitates overcoming ‘perceived barriers’ (HBM). These four domains collectively provide a comprehensive assessment of exercise compliance, from intention to action and maintenance.

The most significant unique contribution of our scale is the inclusion of the ‘preparation compliance’ dimension, enhancing the specificity for LDH patients with conservative treatment. The item “Whether you have been assigned a personalized functional exercise program by healthcare professionals” reflects the necessity of healthcare professionals developing exercise programs for patients, emphasizing the importance of exercise prescriptions. Previous studies have shown that clear exercise goals are a prerequisite for maintaining good compliance behavior (30, 31). This finding is strongly supported by our results. Only by providing detailed plans for patients can the enhancement of the patient’s health beliefs be better achieved. The item “begin functional exercise as soon as the condition allows” follows the concept of fast recovery. Additionally, the decision to retain item Q3 “avoid bending, excessive lifting, getting cold, wearing tight pants, and wearing high heels as advised by healthcare professionals” after statistical and research team deliberation was clinically and theoretically justified. It directly addresses the HBM’s ‘perceived susceptibility’ and ‘perceived severity’ constructs by measuring the patient’s adherence to avoiding known risk factors, a foundational behavioral change that precedes and enables successful exercise participation.

Core stability training can continuously improve the recruitment ability and fatigue resistance of core muscle groups in patients with low back pain, improve proprioception and balance, and alleviate symptoms of low back pain and functional impairments (32, 33). In this study, an exercise compliance dimension was developed. Through literature analysis and expert group discussions, six core muscle strength exercise programs related to the functional exercise compliance scale were selected as health behaviors, including straight leg raises, bridge exercises, push-ups, reverse fly exercises, supine knee flexion, and knee-hand balance exercises. The specific content of home exercise for patients was detailed, including the exercise content and specific frequency, serving as professional guidance. The exceptionally high internal consistency of this dimension (*α* = 0.947) and the strong factor loadings of its items (e.g., 0.869 for push-ups) suggest that adherence to these prescribed exercises is a unified construct. This implies that patients who comply with one exercise are highly likely to comply with the others, which is a positive indicator for the cohesiveness of the prescribed exercise program and the underlying trait of general exercise adherence in this population.

Patients with low back pain often communicate with therapists or doctors to receive mental and psychological support and maintain the motivation for exercise (34, 35). Moreover, family and social support are essential prerequisites for maintaining good compliance (36). Therefore, the supervision compliance dimension in the developed scale of this study emphasized the supervisory effect of family members and healthcare providers. The strong correlation between supervision compliance and exercise compliance (*r* = 0.743, *p* < 0.001) provides robust empirical evidence for this, underscoring that external support and self-monitoring are critical facilitators of the actual exercise behavior.

Additionally, the recommendation compliance dimension mainly involved measuring the compliance of patients in actively seeking medical help when problems arise during the rehabilitation exercise process. The goal of this dimension is to reduce the negative impact caused by negative emotions, poor medical experiences, and negative attitudes, and better promote patients’ control over their rehabilitation process, as well as enhance self-efficacy, thus maintaining high health beliefs and behaviors, improving compliance. A high score in this dimension reflects a proactive patient who partners in their care. We speculate that this behavior is crucial for long-term adherence, as it allows for the adjustment of exercises before frustration or pain leads to complete abandonment of the regimen.

### 4.3 Clinical and research implications

A key strength of this scale is its discriminant validity, which confirms that the four dimensions, while related, capture distinct aspects of the compliance construct. This granularity allows for a nuanced assessment. For example, a clinician might identify a patient with high exercise compliance but low recommendation compliance—a profile that suggests a risk of persisting with improper technique or pain without seeking guidance. Conversely, a patient with high preparation and recommendation compliance but low exercise compliance might need interventions focused on motivation and overcoming practical barriers. This moves beyond a simple ‘adherent/non-adherent’ binary and enables truly personalized patient feedback and intervention strategies.

### 4.4 Methodological rigor and psychometric properties

To ensure the reliability and scientific validity of the scale, the present study strictly followed the scale development procedure (37), which included steps of literature review, expert review, pilot testing, formal testing, and examination of reliability and validity. After an extensive review of the literature on exercise compliance and group discussion, we constructed the item pool. Subsequently, the scale underwent further selection through the Delphi expert consultation method. The selection of 16 consulting experts in this study strictly adhered to the technical requirements of the Delphi method. The experts were from medical universities and tertiary grade-A hospitals in Zhejiang Province, with high academic levels in the fields of orthopedics, acupuncture, and rehabilitation. Therefore, the selection of consulting experts in this study was professionally authoritative. The participation rates of the two rounds of consulting experts were 88.89 and 100%, indicating a high level of enthusiasm among the experts. The authority coefficient of the two rounds of consultation was 0.985, suggesting that the experts had a good grasp and representativeness in the study of exercise compliance in patients undergoing conservative treatment for LDH. The Kendall’s concordance coefficients for importance in the two rounds were 0.196 and 0.219, and for feasibility were 0.143 and 0.218. The statistical significance was *p* < 0.001, indicating that the opinions of the experts gradually converged, and the consultation results were highly reliable.

Through pilot testing, a comprehensive analysis and linguistic refinement of the scale items were conducted to assess the reliability and readability of the scale. Patient responses to the scale were analyzed using the discrete trend method, the CR method, and the correlation coefficient method. It was determined that the removal of item Q3 should be considered, but the selection of items based solely on this has certain limitations, and clinical needs and theoretical analysis should also be considered (38). Patients had no doubts about reading and understanding the items and were able to complete the questionnaire within 15 min, demonstrating the applicability of the scale to LDH patients. Expert opinions and suggestions ensured the quality of the items. Item selection was based on item discrimination, homogeneity, and independence, further ensuring the quality of scale items and making the content of the compliance scale for the functional exercise of LDH patients with conservative treatment more scientific and rigorous.

Additionally, the validity and reliability of the scale were confirmed through mathematical analysis. The I-CVI ranged from 0.813 to 1.000, and the S-CVI was 0.914, both exceeding 0.8, indicating good content validity of the scale (25). This can also effectively reflect the theme of functional exercise compliance in patients undergoing conservative treatment for LDH. EFA yielded four common factors, with all 20 items having factor loadings above 0.400 and a cumulative variance of 73.578%. The results of CFA indicated a model fit with χ^2^/df = 2.642, RMSEA = 0.069, IFI = 0.929, TLI = 0.910, GFI = 0.928, and RMR = 0.061. The correlation analysis revealed coefficients ranging from 0.424 to 0.743 between the dimensions, and from 0.675 to 0.927 between the dimensions and the total scale. These findings suggest strong consistency and alignment between the four common factors and the concept of the total scale, while also highlighting unique differences that indicate a lack of interchangeability (28). Therefore, the scale demonstrates good construct validity. The Cronbach’s *α* coefficient for the scale in this study was 0.942, and the split-half coefficient was 0.876, indicating good internal consistency (27). The test–retest reliability of the scale was 0.901, demonstrating good stability over time (28). Through the validation of reliability and validity, it is concluded that the dimension clarity and item suitability of the functional exercise compliance scale for patients undergoing conservative treatment for LDH developed in this study are good, with satisfactory reliability and validity.

### 4.5 Limitations and future directions

This study has several limitations. First, the use of convenience sampling from a single tertiary hospital may limit the generalizability of our findings to broader populations and other healthcare settings. Future multi-center studies employing random sampling are needed to validate the scale’s applicability. Second, the cross-sectional design precludes assessment of the scale’s responsiveness to change over time or following interventions. Establishing the minimal clinically important difference through longitudinal studies is an important next step. Third, while missing data were minimal (<5%) and unlikely to bias results, we did not employ advanced statistical methods to handle them. Such methods (e.g., multiple imputation) should be considered in future work with larger missing data. Finally, as a self-report measure, the scale may be susceptible to social desirability bias. Although this is common to all adherence scales, supplementing self-report with objective measures (e.g., wearables) in future research would strengthen findings. Despite these limitations, this study provides a validated tool for a previously unmeasured construct. Addressing these points in future research will further enhance the scale’s utility.

## 5 Conclusion

The functional exercise compliance scale for LDH patients with conservative treatment, developed in this study, consists of 4 dimensions and 20 items. This scale, with a unique four-dimensional structure, particularly the novel preparation compliance dimension, fills an important measurement gap by providing the first specific tool for conservatively managed LDH patients. This scale holds significant scientific and practical value, as it serves as a vital tool for healthcare professionals and patients to consistently and actively evaluate compliance with functional exercises. Patients are considered both participants and supervisors of functional exercise in this scale. They can identify weaknesses in their exercise routines, motivating them to seek assistance from healthcare providers. Furthermore, it aids healthcare providers in assessing and continuously monitoring patients’ compliance with exercises over time. When monitoring patients engaged in home exercise programs, establishing the minimal parameter for score fluctuations is crucial. This ensures the accuracy and reliability of research findings while reducing variations caused by different measurement tools. We believe that this scale holds substantial promise for improving rehabilitation quality and long-term patient outcomes in the conservative management of LDH.

## Data Availability

The raw data supporting the conclusions of this article will be made available by the authors, without undue reservation.
